# Pediatrics

**Published:** 1896-01

**Authors:** Maud J. Frye

**Affiliations:** Clinical instructor in diseases of children, Medical Department, University of Buffalo


### PEDIATRICS.

REPORTED BY MAUD J. FRYE, M. D.
Clinical instructor in diseases of children, Medical Department, University of Buffalo.

### PYGOPHAGUS.

DR. JACOBI (*Archives of Pediatrics*, Oct., 1895,) reports a case occurring in the practice of Dr. Sigmund Tynberg. The term is applied to a double monster, consisting of two complete individuals united in the region of the sacrum or coccyx. The sex in this case was female. They were united below the third sacral vertebra, the sacra united on one side. Each child had its own complete generative organs and each its distinct circulatory apparatus. The anus was common, the rectum divided by an antero-posterior septum. The spinal cords did not communicate. The twins lived nearly five months, one dying eight hours before the other. Only thirteen similar cases are on record.

---

### NASAL CATARRH.

BERGMANN (*Archives of Pediatrics*, Oct., 1895,) treats the noses of children, even infants, suffering from a cold in the head, after the plan of Henoch, applying a 2 per cent. solution of silver nitrate with a brush. The following day the application may be repeated. The solution has always proved inoffensive and will, he believes, do much toward preventing deflected septum and hyperplasia of the post-nasal adenoid tissue.

---

### A DANGER OF PASTEURISED MILK.

*Archives of Pediatrics* (Sept., 1895,) editorially says: A most important detail is the rapid cooling after the application of heat.

A temperature of 167° F. kills all pathogenic germs, but it does not kill their spores. If the milk, after heating, is not properly cooled and is allowed to stand in a warm place, it may remain at a temperature exactly adapted to the growth and development of these spores. The process thus becomes a source of danger rather than a safeguard.

---

### MODIFIED MILK.

Worcester (*Boston Med. and Sur. Jour.*, Sept. 19, 1895,) gives the Dresden modification of cow's milk as follows : white of one egg, one pint of cow's milk of 9½ per cent. richness in fat, one and one-half pints of water, thirteen drams of milk sugar. Sterilise milk before adding other ingredients. Sterilise milk sugar in a sealed jar. All articles used in preparing the food should be sterile. Wash the egg in absolute alcohol, break, and stir the milk sugar slowly into the white. Add the water slowly and strain into the milk, previously cooled to below 100° F. Pour into bottles and stopper with sterile cotton. With care in preparation, the mixture will keep for days. The egg supplies a substitute for lact-albumin, which the ordinary modifications lack.

---

### MATERNAL FACTORS IN RICKETS.

Garrod and Fletcher (*Brit. Med. Jour.*, Sept. 21, 1895,) enumerate as the most important maternal factors in the causation of rickets the following :

Antepartum : (1) Ill-health, malnutrition or disease of mother during pregnancy. Phthisis concurrent with pregnancy is a most important factor. (2) Want of fresh air and exercise during pregnancy. (3) Numerous and rapid pregnancies. (4) Multiple pregnancies. (5) Age of mother at birth of child. A mother advanced in life is more apt to produce rickety children than a younger woman. Postpartum or location factors are : (1) Deficiency of milk in quantity or quality. (2) Mother's health during lactation, menstruation and pregnancy occurring during lactation. (3) Over-lactation.

Cases illustrating the influence of these several factors are cited. In many cases many of the maternal factors may come into play in causing the disease. These factors have an important bearing upon the prevention and treatment of the disease, although obviously some are beyond the control of the medical attendant.

### THE LOCAL TREATMENT OF THE SKIN.

SEIBERT (*Archives of Pediatrics*, Sept., 1895,) has observed beneficial effects from the use of 5 per cent. ichthyol ointment in cases of erythema nodosum, peliosis rheumatica, scarlatina and measles. In scarlatina the change for the better is striking. The swollen red skin shrinks and turns pale brown, the temperature declines three to four degrees within a few hours, and the nervous, itching, peevish child becomes quiet, better humored and usually goes to sleep without coaxing.

### ENTERIC FEVER IN INFANCY.

NOYÈS, (*Journal Am. Med. Ass'n*, Sept. 28, 1895,) in a valuable article on the subject, says: Typhoid fever occurs in infancy very commonly in epidemics, also sporadically where possibly the disease is overlooked. The pathologic changes in infantile cases, while distinct, are less severe than in adults. There is generally no ulceration of Peyer's patches.

The diagnostic symptoms characteristic of infancy may be briefly stated as follows:

1. Any long-continued fever which does not yield to quinine, especially if of gradual onset with no existing condition of throat, lungs or bowels to offer an explanation. If the continuous fever is well borne it is still more suggestive of typhoid.

2. The lack of acute symptoms of an intestinal nature is more characteristic of typhoid fever than are violent symptoms. Constipation is noted in many cases to be succeeded by diarrhea at the end of the first week. Sordes is far less frequent than in adults.

3. Rose spots are to be expected in most cases.

4. Symptoms of headache frequently occur.

5. An enlarged and painful spleen is an important symptom.

6. Tympanitis in most cases is moderate.

7. Bronchitis is as regular a symptom as in measles.

8. Angina may occur and will confuse the diagnosis.

9. Epistaxis and hemorrhage from the bowels are both rare.

10. The Eberth bacillus in the stools has not been positively demonstrated. The bacilli can frequently be found in the lymphatic tissues and the spleen. In a few cases they have been found in the blood, the urine and the buccal secretions.

11. Erlich's test, which is yielded only by typhoid, miliary tuberculosis, septicemia and cancerous cachexia, may assist the diagnosis.

The disease appears in three forms: Abortive, of short duration; a type resembling the ordinary adult type and a malignant form. Relapses are infrequent and convalescence, as a rule, is quicker and less complicated. The malignant form may be characterised by symptoms which simulate meningitis. In nearly all cases of this nature, simple hyperemia is all that the autopsies develop. The disease is to be looked for in this country in a mild or abortive form. A severe and prolonged case may be the result, not of simple infection by the typhoid bacillus, but a complication by other pathogenic bacteria.

---

### ANTITOXIN IN DIPHTHERIA.

THE *University Medical Magazine* (Nov., 1895,) says: A careful weighing of all the evidence submitted upon the subject up to the present time seems to establish the following facts: that antitoxin is a curative agent far more efficacious in diphtheria than any remedy heretofore employed; that its injection is very rarely followed by serious local disturbances, such as abscess, and, perhaps, never when the preparation is pure and employed under antiseptic precautions; that a marked improvement in both the local and general symptoms of diphtheria is noticeable within twenty-four to forty-eight hours after the injection of the serum; that the antitoxin has a decided influence in preventing the spread of the false membrane into the larynx and trachea; that the earlier in the course of the disease the serum is employed the more favorable are the results; that it is distinctly more efficient in the fibrinous types of the disease than in the septic ones; that the liability to paralysis and albuminuria is not lessened by the use of serum, but, probably, somewhat enhanced thereby; that genuine nephritis, on the other hand, is less frequently observed in cases of diphtheria treated with antitoxin than with other remedies; that antitoxin may produce certain untoward symptoms, such as various cutaneous rashes, but that these are not serious in their nature and are unattended with danger to life; and that improvement in the methods of preparing the serum and more definite knowledge as to the manner of its employment have rendered the later reports even more favorable to its use than the earlier ones.

### HIGH MORTALITY AMONG THE NEWLY-BORN.

CROCKETT (*Medical News*, August 3, 1895,) concludes: (1) The mortality of the newly-born is both absolutely and relatively disgracefully high. (2) Septic infection causes a part of this mortality, and is preventable. (3) Asepsis should be observed about the new-born child. Dirty fingers or instruments should not be introduced into the child's mouth in cases of asphyxia. The child should not be allowed to ingest pus with its milk. (4) The navel should be treated on modern surgical principles and have the personal supervision of the physician. (5) The physician should take the child's temperature as well as the mother's, bearing in mind that many of the septic diseases have an insidious approach, and that the importance of the thermometer cannot be overestimated. (6) The navel wound may be the path of infection without showing signs of local inflammation. (7) Close observation of the new-born infant will teach the physician much and benefit the child greatly.

